# Siphon-Controlled Automation on a Lab-on-a-Disc Using Event-Triggered Dissolvable Film Valves

**DOI:** 10.3390/bios11030073

**Published:** 2021-03-06

**Authors:** Brian D. Henderson, David J. Kinahan, Jeanne Rio, Rohit Mishra, Damien King, Sarai M. Torres-Delgado, Dario Mager, Jan G. Korvink, Jens Ducrée

**Affiliations:** 1 School of Physical Sciences, Dublin City University, Dublin 9, Ireland; Brian.Henderson@tudublin.ie (B.D.H.); jeanne.rio02@gmail.com (J.R.); 2 School of Mechanical Engineering, Dublin City University, Dublin 9, Ireland; david.kinahan@dcu.ie; 3 National Center for Sensor Research, Dublin City University, Dublin 9, Ireland; 4 I-Form, the SFI Research Centre for Advanced Manufacturing, Dublin City University, Dublin 9, Ireland; 5 The Water Institute, Dublin City University, Dublin 9, Ireland; 6 Fraunhofer Project Center, Dublin City University, Dublin 9, Ireland; rohit.mishra@dcu.ie (R.M.); damien.king@dcu.ie (D.K.); 7 Institute of Microstructure Technology, Karlsruhe Institute of Technology, 76344 Karlsruhe, Germany; sarai.delgado@kit.edu (S.M.T.-D.); dario.mager@kit.edu (D.M.); jan.korvink@kit.edu (J.G.K.)

**Keywords:** lab-on-a-chip, centrifugal microfluidics, lab-on-a-disc, siphon valves, cardiovascular disease, chemiluminescence, immunoassay

## Abstract

Within microfluidic technologies, the centrifugal microfluidic “Lab-on-a-Disc” (LoaD) platform offers great potential for use at the PoC and in low-resource settings due to its robustness and the ability to port and miniaturize ‘wet bench’ laboratory protocols. We present the combination of ‘event-triggered dissolvable film valves’ with a centrifugo-pneumatic siphon structure to enable control and timing, through changes in disc spin-speed, of the release and incubations of eight samples/reagents/wash buffers. Based on these microfluidic techniques, we integrated and automated a chemiluminescent immunoassay for detection of the CVD risk factor marker C-reactive protein displaying a limit of detection (LOD) of 44.87 ng mL^−1^ and limit of quantitation (LoQ) of 135.87 ng mL^−1^.

## 1. Introduction

Cardiovascular disease (CVD) encompasses a large array of disorders. The most common is arteriosclerosis which is the buildup of plaque on the arterial wall, restricting the flow of oxygen to different tissues in the body. CVD is among the primary causes of death worldwide with an estimated 15.5 million incidences in 2012 and is becoming more prevalent with increasing standards of living across the global population [1]. Heart failure has emerged as a particularly prevalent diagnosis in sub-Saharan Africa [2], but other often under-researched conditions, which are relatively limited to tropical regions, such as endomyocardial fibrosis (EMF) and rheumatic heart disease, are becoming more prevalent [1]. Among mitigation strategies proposed to address these issues are the implementation of field-friendly Point-of-Care (PoC) technologies to assist diagnosis [1,3]. To address this need, lab-on-a-chip devices can provide bioanalytical tests on patient samples [4,5,6,7].

A wide range of biomarkers have been associated with CVD, including creatine kinase (CK), creatine kinase-MB (CKMB), myoglobin, and C-reactive protein (CRP) as well as cardiac troponin I and T. Typically, after a cardiovascular event that restricts blood flow to the tissue, these molecules are released into the blood from dying cells. However, as these molecules can also be present in other tissues, there can be considerable variability in predictive power of these markers for CVD [8]. Of identified biomarkers, CRP has been assigned as the inflammatory biomarker with the highest relevance for clinical practice by the American Heart Association and American Center for Disease Control [9].

The association between C-reactive protein and cardiovascular events comes from the activities of necrotic tissue [10]. In case such tissue becomes damaged, for example, when oxygen-rich blood is restricted from reaching the heart (acute myocardial infarction (AMI)), the affected cells release cytokines to recruit macrophages to the affected area. As part of the defense system, these macrophages perceive this activation as evidence of a pathogenic invasion, which then causes the production of interlukin-6. Interlukin-6 is also released from vascular smooth muscle cells during the formation of atherosclerosis, which is a precursor to AMI and other cardiovascular events [11].

Its indirect link between cardiovascular events creates an opportunity to use CRP levels in plasma as a biomarker for cardiovascular disease. The levels of CRP in patient plasma can be affected by inflammation of coronary vessels by pathogen infection, inflammation related to the atherosclerotic process, extent of myocardial ischemia, and the amount of free-circulating pro-inflammatory cytokines [10]. Levels of CRP in healthy individuals are usually below 10 μg mL^−1^. However, during an inflammation response, these levels can rise quickly by three orders of magnitude [9,10]. It has also been found that increased levels of CRP have been identified as a prognostic biomarker for cardiovascular events due to its association with arteriosclerosis [9,10].

Thus, there exists a need for a lab-on-a-chip device that can provide distributed point-of-care detection of CVD-related markers, such as CRP, in low-resource settings. Particularly, such a device should meet the World Health Organization ASSURED criteria being Affordable, Sensitive, Specific, User-friendly, Rapid/Robust, Equipment-free, and Deliverable to end users. A technology platform that offers great potential to address the requirements of biomedical point-of-care testing is the centrifugal microfluidic platform, or lab-on-a-disc (LoaD) [12,13,14,15,16,17]. The LoaD platform is often based on the geometric footprint similar to the 120-mm diameter Compact Disc™ (CD) or Digital Versatile Disc™ (DVD) common in optical data storage. This offers the potential to scale up to mass manufacture through adaptation of established fabrication processes. These disc-shaped cartridges are typically rotated about their axis on a relatively low-cost spindle motor (often referred to as centrifugal test-stand or ‘spin-stand’). Within biomedical testing, the LoaD platform has been used to automate a range of common test formats, including small-molecule assays [18], nucleic acid purification and amplification [19,20], and various types of immunoassays [21,22]. The LoaD platform has also been used to automate emerging diagnostic tests such as cell-free DNA [23] and quantification of circulating tumor cells [24,25]. The LoaD platform has also been applied to other application fields such as environmental monitoring [26,27,28].

Compared with other microfluidic lab-on-a-chip technologies, the LoaD offers several decisive advantages. In the first case, the ability to centrifugally sediment material during sample preparation is a major asset, particularly for sample preparation of blood [29] and preconditioning other non-homogenous samples such as milk [30,31]. Another key advantage is its capability to transfer a biological assay from ‘wet bench’ to ‘on chip’ with minimal modification. The LoaD platform provides a number of robust and high-performance laboratory unit operations (LUOs) [13] such as metering [32], aliquoting [33], mixing [34], and routing [35]. This enables automation of the vast majority assay protocols that are conducted on the wet bench using a pipette and centrifuge. Furthermore, LoaD systems do not require expensive and specialized microfluidic pumps and, importantly, they can be loaded via pipette, syrette, or swab directly and without complex and error-prone priming procedures [36]. 

Automation of protocols on the LoaD is highly dependent on reliable flow-control/valving technology. Broadly speaking, these valving strategies can be divided into fully rotationally controlled (passive) and instrument-supported/externally actuated (active) schemes. Active schemes have recently been reviewed [37]. Passive valves depend on the geometry or features of the disposable LoaD cartridge. They are opened by modulating the spin rate, with a geometry-dependent threshold frequency for opening. Active valves rely on interaction with an instrument-based unit (often integrated into the centrifugal spin-stand). A third, less common, valving technology is based on the timed disintegration of (water) dissolvable films (DFs) and/or liquid movement [38,39]. A comparison of different valving technologies, particularly as related to passive flow-control and to the dissolvable film valves used in this study, is provided in Table 1. 

The focus of this work was an advancement of the event-triggered dissolvable films valves (Table 1), whereby their performance is enhanced through coupling them with a centrifugo-pneumatic siphon valve. Event-triggered valves function akin to single-use electrical relays. The arrival of liquid at a first DF, referred to as the control film (CF), triggers the release of a liquid at a second DF at a distal location, referred as the load film (LF). Properly configured, this coordinates the release of liquid bioreagents in a well-defined sequence. This valving technology offers independence from the spin rate (as valve actuation is governed by DF dissolve time and liquid movement) and the limiting factor on the number of LUOs/valves in sequence is only the available space on the disc. 

A critical limitation of event-triggered valving is that timing of their actuation depends on the dissolution time of the DFs and intervals for liquid transfer about the disc. While their dissolution time can be tailored by their formulation [62,63,64] and even used for reagent storage [65,66], resorting to commercially available (single-grade) DFs in microfluidic devices is beneficial toward mass manufacture [67]. To control timing of event-triggered valves, researchers have focused on alternative mechanisms such as hybrid event-triggered valves that are opened using supporting instrumentation [61]. Another approach, and the focus of this article, is to place a reusable and rotationally controlled valve (i.e., a siphon valve [45] or centrifugo-pneumatic siphon valve [50,51]) in the flow path for on-disc flow control. This strategy allows the disc architecture to govern the sequence of sample/reagent release while changes in spin rate control the timing of reagent release. This platform establishes arbitrarily defined (i.e., programmable via spindle motor) incubation periods that are often required in typical assay protocol.

Chemiluminescence (and chemiluminescent immunoassays) have been previously demonstrated within lab-on-a-chip devices [68,69,70,71,72,73] and, specifically, on LoaD platforms [9,74,75,76,77]. In this work, we demonstrated detection of CRP, from buffer, using a chemiluminescent immunoassay in the clinically relevant range. Note that in many assays, such as the one described here, samples are diluted in dilute inhibitory proteins/molecules. In the case of CRP, concentrations in blood are typically in the μg mL^−1^ range but, due to the dilution associated with this assay, the detection levels measured are in the range of ng mL^−1^. 

The fluidic architecture presented in this manuscript, which combined a centrifugo-pneumatic siphon valve with event-triggered valves, unprecedentedly demonstrated full fluidic control, with arbitrarily timed incubations, of eight different reagents. Using off-disc measurements, our system showed a linear relationship (R^2^ = 0.91) between concentration and luminescent intensity in a range of measurements (*n* = 3) made between 0 to 80.5 ng mL^−1^ The limit of detection was 29 ng mL^−1^, which represents patients at the threshold of the high-risk category of cardiovascular disease.

## 2. Materials and Methods

### 2.1. Disc Architecture

**Figure 1:**
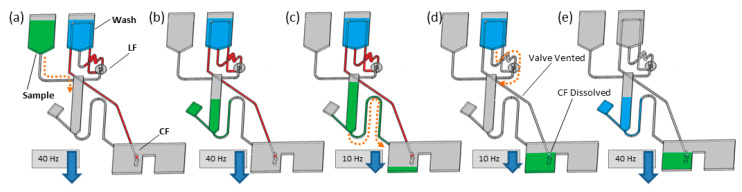
Event-triggered sequential release of centrifugo-pneumatic siphon valves (CPSVs). (**a**,**b**) Loading of sample (green), at a high spin rate, into the incubation chamber. Alternating the spin rate can be used to induce mixing in the incubation chamber. (**c**) At a low spin rate, the centrifugo-pneumatic siphon valve is primed and (**d**) then emptied at a medium spin rate. (**e**) The control film (CF) of the following valve is wetted to vent the pneumatic channel release to the next reagent (blue). This process can be repeated to control the timed release and incubation of further reagents.

**Figure 2:**
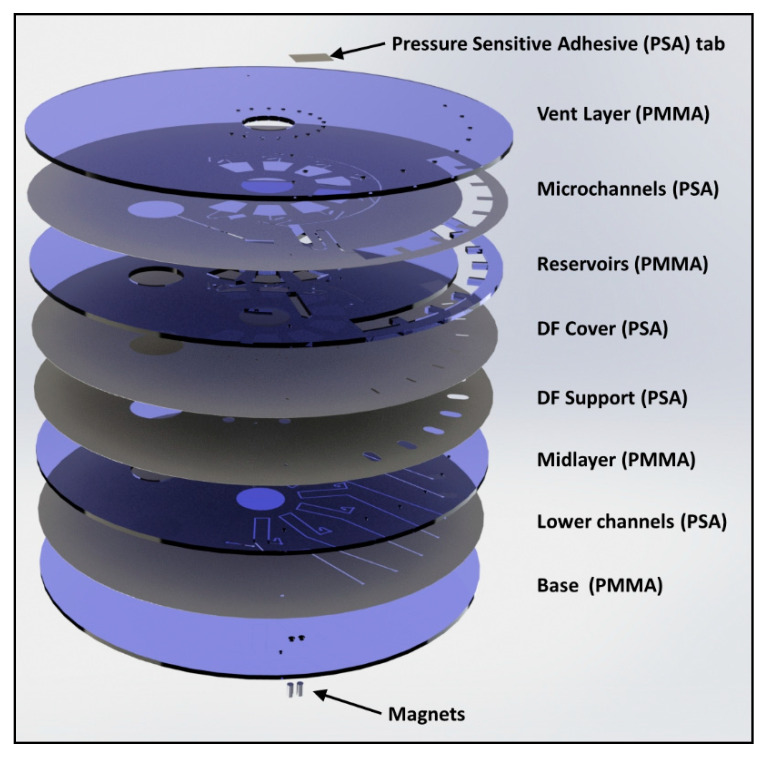
Multilayer assembly of the chip. The PSA tab covers a sample removal hole in the vent layer during disc operation. The magnets are held in place by an interference fit and hold the paramagnetic beads in place within the disc.

**Figure 3:**
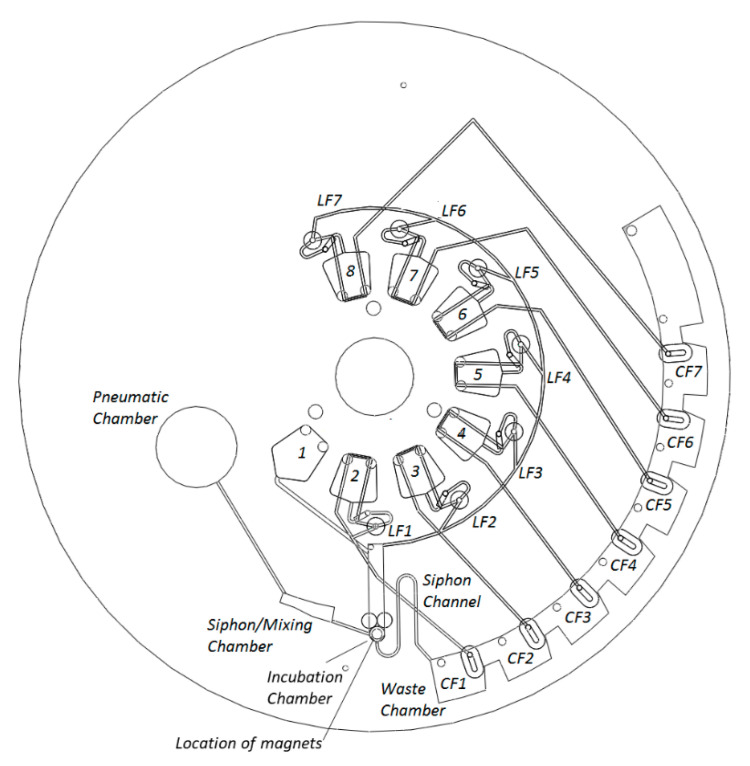
Disc architecture. Reservoir 1 (R1) contains magnetic beads in buffer, which are held inside the reaction chamber. These beads have been prepared off-chip with the capture antibody. R2 contains the sample, R3 and R4 are filled with washing buffers, R5 contains the detection antibody, R6 and R7 contain wash buffers, and R8 contains the substrate to catalyze chemiluminescent detection. Note, for data presented in Figure 6, R8 was left empty and, following the final washing step (R7), the disc was stopped. The magnets were removed, and the beads were suspended in buffer and aspirated into a pipette through a vent on the incubation chamber. Off-disc measurement was made via GloMax 96 Microplate Luminometer according to the protocol described in ESI.

**Figure 4:**
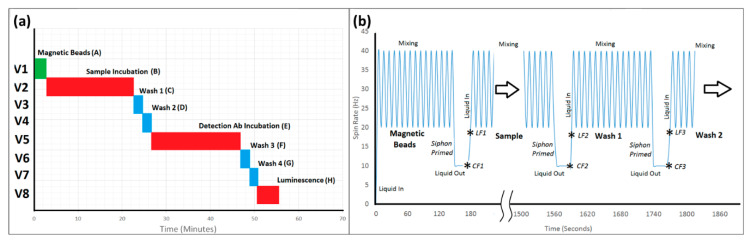
(**a**) Chemiluminescent immunoassay protocol with incubation times. (**b**) Typical spin protocol describing the first three LUOs automated on the disc. Note that the protocol was fully automated using a programmable, human-readable macro integrated with the centrifugal test stand using LabVIEW.

In this paper, we demonstrate robust, highly multiplexed rotational flow control of one capillary valve and seven event-triggered DF valves. Incubation intervals are defined by changes in the spin rate of a programmable spindle motor. This was enabled by placing an incubation chamber, gated by a centrifugo-pneumatic siphon valve, between the central reagent storage reservoirs and the liquid waste chamber (Figure 1, Figure 2 and Figure 3). At high spin rates (in this case 40 Hz), the liquid (i.e., sample, reagents, or wash-buffer) enters the incubation chamber and is retained by the unprimed siphon valve. As typical for CPSV valves, liquid is displaced into a dead-end pneumatic chamber, which ensures that the liquid level remains below the siphon crest. As the incubation chamber retains the liquid, downstream CFs are not yet wetted. The spin rate can be varied in the interval between 20 Hz and 40 Hz, which displaces liquid into the incubation chamber and enhances mixing, without priming the siphon valve. Following incubation, the durations of which are described in Figure 4, the spin rate is reduced to 10 Hz, which expels liquid from the pneumatic chamber and increases the liquid level in the incubation chamber. The siphon valve then primes, and liquid is transferred into the waste chamber.

The waste chamber is segmented into volumes that are equal or slightly less than the volume of liquid released from the incubation chamber. Each segment of the waste chamber contains a CF, which, when wetted, will open an event-triggered valve. Thus, the transfer of liquid to the waste chamber prompts the release of the next liquid defined in the protocol. This combination of the event-triggered valving with the centrifugo-pneumatic siphon enables arbitrarily timed reagent delivery and so establishes freely definable incubation intervals. 

### 2.2. Disc Manufacture and Assembly

The discs were designed with SolidWorks (Dassault Systèmes, Paris, France) as a 3D structure and then 2D AutoCAD Drawing Exchange Format (AutoCAD DXF) files were extracted from this model (Figure 2). Individual layers were then machined based on these drawings. Poly-(methylmethacrylate) (PMMA) layers were machined from 1.5-mm-thick PMMA sheets (Radionics, Ireland) using CO_2_ laser ablation (Exilog Zing, Golden, CO, USA). Medical-grade Pressure Sensitive Adhesive (PSA) (ARCare^®^ 7840, Adhesives Research, Limerick, Ireland) was structured using a knife-cutter (Graphtec CE6000-40, Irvine, CA, USA). The disc was assembled (Figure 2) from a stack of eight layers:

(1) Vent layer of PMMA, containing loading ports/air vents;
(2) Microchannel layer of PSA, containing microchannels for reagent and air transport;
(3) Reservoir layer of PMMA, containing reagent reservoirs, waste chambers, pneumatic chambers, incubation chamber, and connecting vertical vias;
(4) DF cover layer (PSA), which seals DF tabs into the disc;
(5) DF support layer (PSA), which provides alignment and mechanical support for DF tabs;
(6) Intermediate layer (PMMA) provides mechanical support for DFs;
(7) Lower channels (PSA), containing microchannels for reagent and air transport; and
(8) Base (PMMA) provides a layer to seal the lower channels. This layer also contains mechanical support for permanent magnets.

In addition to these layers, permanent magnets (S-03–06-N, Supermagnete, Gottmadingen, Germany) are embedded (mechanical fit) into the base layer to provide a point of agglomeration for the paramagnetic beads. Additionally, a loading hole for reagent removal (located in the vent layer) is sealed with adhesive tape. DF tabs were assembled, as described previously [38], from gas-tight, water-dissolvable film (KC-35, Aicello, Aichi, Japan). These tabs take approximately 30–40 s to dissolve in water at room temperature [38] and provide a short time delay between wetting of the DFs in the waste chamber and release of the next reagent.

The layers from which the cartridges were assembled were cleaned prior to assembly. This protocol includes washing the PMMA in dilute Micro-90^®^ Concentrated Alkaline Cleaning Solution (International Products Corporation, Burlington, NJ, USA) twice (20-min sonication at 60 °C), followed by washing in deionized (DI) water twice (20-minute sonication at 60 °C), and air drying in a HEPA-filtered assembly room [50,59]. PSA layers were prepared for assembly under sterile conditions; here, the liners are only removed in a High-efficiency particulate air -filtered (HEPA-filtered) cleanroom. The discs were assembled manually on a custom alignment jig. Between addition of each layer, the discs were rolled at least 12 times (three times and four orientations) using a high-pressure laminator (HL-100, Cheminstruments, Fairfield, OH, USA).

### 2.3. Centrifugal Test Stand

**Figure 5:**
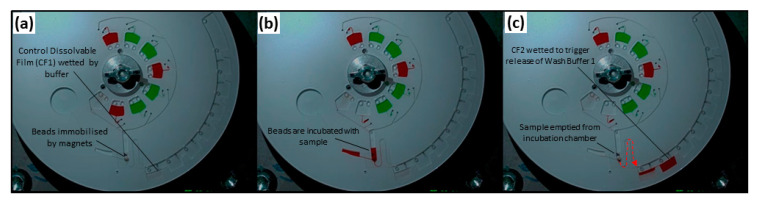
Images acquired using stroboscopic test stand with dyed water representing reagents. (**a**) Magnetic beads are loaded into the chamber and secured via magnetic force in the incubation chamber. (**b**) Red dye solution, mimicking the sample, is released through a DF valve into the incubation chamber. (**c**) The ‘sample’ is transferred to the waste chamber where wetting the next DF releases the first wash buffer.

The discs were characterized on a centrifugal ‘test stand’ [29]. Here, a computer-controlled spindle motor (FESTO, Esslingen, Germany) was synchronized with an externally triggered CCD camera (Pixelfly, PCO, Kelheim, Germany) and strobe light (BVS II, Polytec, Waldbronn, Germany) so that each image was acquired at the same angular position. Therefore, the disc appeared stationary while rotating at even up to 60 Hz. The motor was controlled by custom software (LabVIEW), which enabled programming a spin profile (Figure 4 and Figure 5).

### 2.4. Biological Assay Materials

The CRP capture antibody, rabbit polyclonal to C-reactive protein, was purchased from Abcam plc (ab31156, Abcam plc, Cambridge, UK). The detection antibody used was a Horse Radish Peroxidase (HRP), labelled goat polyclonal anti-CRP (PA1-28329, Thermo Fisher, Dublin, Ireland). Note that polyclonal antibodies were chosen over a monoclonal antibody in order to ensure greatest chance of assay success (with minimal knowledge of protein structure) while accepting a decrease in assay sensitivity (compared to monoclonal antibodies). The 2.8-μm superparamagnetic beads (Dynabeads^®^ M-270 Epoxy, Thermo Fisher, Dublin, Ireland) and reagents required for coupling the capture antibody to the beads were acquired as part of the Dynabeads Antibody Coupling Kit (14311D, Thermo Fisher, Dublin, Ireland). The protein standards used were from the CRP Human Kit for Luminex^®^ Platform (Catalogue number: LHP0031, Thermo Fisher, Dublin, Ireland). Additional reagents used were taken from the Human Extracellular Protein Buffer Magnetic Reagent Kit Thermo Fisher, Dublin, Ireland, Catalogue number: LHB0001). The substrate used was Pierce™ ECL Western Blotting Substrate (T Thermo Fisher, Dublin, Ireland, catalogue number: 32106).

### 2.5. Dynabead Antibody Coupling Procedure

Conjugation of primary antibody to Dynabeads^®^ M-270 Epoxy was performed as per the manufacturer’s instructions. Final concentration of primary antibody (ab31156, Abcam plc, Cambridge, UK)-coated Dynabeads^®^ in solution was 10 mg/mL with each mg of beads coated with approximately 1 µg of antibody. See also ESI.

### 2.6. Benchtop Magnetic Chemiluminescence Assay

**Figure 6:**
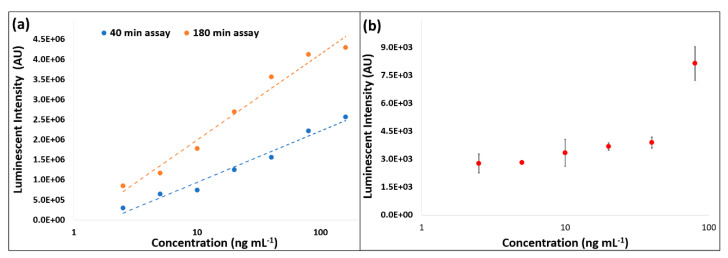
(**a**) Benchmark (on-bench) magnetic chemiluminescence assay showing the standard 180-minute protocol (orange) and the optimized/accelerated 40-minute assay (blue). (**b**) Measurements (arbitrary units) from the on-disc CRP assay (*n* = 3 at each data point). As described in Materials and Methods, all incubation and washing steps took place on-disc, except detection (addition of HRP). Beads were pipetted from the disc and chemiluminescence to a benchtop reader (GloMax 96 Microplate Luminometer).

The assay was optimized on-bench with a particular focus on reducing the assay time. The total assay time was reduced from 180 minutes (according to the recommended protocols) to 40 min (Figure 6a). 

Briefly, all standards were made by diluting CRP stock (CRP Human Kit for Luminex^®^ Platform (Catalogue number: LHP0031, Thermo Fisher, Dublin, Ireland)) with 1X Incubation buffer (Human Extracellular Protein Buffer Reagent (Catalogue number: LHB0001), Thermo Fisher, Dublin, Ireland) to the appropriate concentration. Then, 20 μL of primary antibody-coated Dynabeads^®^ (10 mg/mL) were placed in each well of a white, 96-well plate. Using a small neodymium magnet, the Dynabeads were held in place while the liquid was removed and discarded. Each well was then blocked, using a 1% solution of BSA in 0.1 M PBS for 1 h at 4 °C. As before, the contents of the well were discarded while the magnetic microparticles were held in place with a magnet. Then, 100 μL of each CRP standard was added to the corresponding well and incubated at room temperature for a given incubation time (standard incubation 2 h). 

Each well and microparticle content were then washed twice with 200 μL of wash solution (Human Extracellular Protein Buffer Reagent (Catalogue number: LHB0001), Thermo Fisher, Dublin, Ireland). Then, 200 μL of detection antibody (PA1-28329, Thermo Fisher, Dublin, Ireland) (1/10,000 dilution in 0.1M PBS) was added to each well and incubated for a given incubation time in the dark (standard incubation 1 h). Each well and microparticle contents were then washed twice with 200 μL of wash solution, as before. Just prior to plate reading, the final wash solution was removed from each well and 100 μL of Pierce™ ECL Western Blotting Substrate (Catalogue number: 32106, Thermo Fisher, Dublin, Ireland) was added to each well. Wells were read using a GloMax 96 Microplate Luminometer. See also ESI.

### 2.7. Lab-on-a-Disc Magnetic Chemiluminescent Assay 

Prior to their loading to the disc, the magnetic beads were prepared on-bench with the capture antibody according to the protocols described in Section 2.5.

Prior to being placed on the centrifugal test stand, each disc cartridge had two neodymium magnets placed in precut holes behind the incubation chamber. Next, each reservoir was loaded with 90 µL of a specific reagent, corresponding to a step of generic immunoassay. On-disc reservoirs were filled according to Table 2. R2 contained the specific CRP standards at differing concentrations (80, 40, 20, 10, 5, or 2.5 ng mL^−1^), depending on the concentration of standard being tested. R8 was left empty during assay testing to enable off-disc measurement. See also ESI.

### 2.8. Automated Lab-on-a-Disc Protocol

A pre-programmed protocol was executed unsupervised on the centrifugal test stand. Mixing cycles refer to the number of times the disc was accelerated between 20 Hz and 40 Hz to induce advective mixing. Note the chemiluminescence substrate was not loaded on-disc.

After completion of the automated spin protocol, the disc was removed from the test stand and the magnets were removed from the underside of the disc. Next, the scotch tape was stripped from over the incubation chamber. Then, 60 μL of 0.1M PBS was pipetted into the incubation chamber and the magnetic beads were suspended in the buffer. The buffer and beads were then pipetted from the disc and transferred to a well plate. A magnet retained the beads while supernatant was extracted. Next, 90 μL of 0.1M PBS was loaded into the microtiter plate for resuspending the beads. Under low-intensity illumination, 100 μL of Pierce™ ECL Western Blotting Substrate reagent was added to each the well on the well plate and chemiluminescent signal was measured on a luminometer (GloMax Microplate Luminometer, Promega, Madison, WI, USA).

## 3. Results

Images of the operation of this microfluidic architecture, acquired from the test stand, are shown in Figure 5. Video showing the full sequence of valve actuation (albeit, with shortened incubation times and using colored water for better visualization) is provided in ESI Video S1 and ESI Video S2. Videos were post-processed as described in ESI. As can be seen in Figure 5, this microfluidic architecture allowed complete control of incubation times through altering the spin rate to open the valves. The architecture proved to be extremely reliable. Once the manufacturing process was established, 18 of 21 discs functioned as expected under automated control of the spindle motor (Figure 6b). The on-disc CRP assay was completed (*n* = 3) at six different concentrations and a limit of detection (LoD) of 44.87 ng mL^−1^ and limit of quantitation (LoQ) of 135.87 ng mL^−1^ were established.

## 4. Discussion and Conclusions

The efficacy of this disc architecture to implement paramagnetic bead-based chemiluminescent immunoassays for CRP was established. The clinical range of interest for CRP is between 3–80 μg mL^−1^ for adults, and, in the case of neonatal sepsis, as low as 0.08 μg mL^−1^ [9]. In this work, we demonstrated the capability to detect as low as 44.87 ng mL^−1^ in buffer. Typically, in a diagnostic test, plasma or serum is isolated from whole blood and then diluted by a defined ratio. This ensures the assay can deliver results in the clinically relevant range while not delivering a signal at saturation. This dilution also improves the accuracy of the assays by diluting components of blood, which may inhibit the assay performance. Implementing this full protocol on disc, including dilution of sample, will be subject to future work. Indeed, integration of this microfluidics architecture with wireless chemiluminescent sensing [74] offers the potential to provide for full sample-to-answer automation of the assay on a compact laboratory instrument.

Figure 6b shows measurements across a range of concentrations with both standard and optimized incubation times. These parameters were then used on disc and reduced the overall assay time from over 2 h to approximately 55 min. These same incubation times were then used for the on-disc protocol. However, while measured on the same laboratory instrument, the signals measured from the beads processed on disc were significantly lower than those processed on bench (Figure 6).

We identified two potential causes of this reduced signal. In the first, the removal of beads from the disc for measurement can result in loss of material. This loss may be mitigated through integration of chemiluminescence measurement into the test-stand instrument [74]. The second problem concerns the use of magnetic beads which, in this implementation, are pulled to the surface by permanent magnets embedded into the underside of the disc. Ideally, this would create a monolayer of functionalized beads to replace an antibody-coated surface [78,79]. A particular advantage of using magnetic beads is the potential to use the same disc architecture against different antibody targets. However, we believe the use of permanent magnets caused unfavorable agglomeration of magnetic beads and, despite rigorous mixing, leaving significant percentage of beads underexposed to sample and/or to detection antibody. This effect may be mitigated through use of an electro-magnet rather than permanent magnets. This would permit the beads to mix freely with the sample/reagents during the incubation steps. Alternatively, the PMMA surfaces inside the disc might be directly functionalized to provide a defined surface for detection [80].

The results presented above clearly demonstrate the capability of this microfluidic architecture to enable biological protocols that require extended incubation periods (i.e., of the order of 20 min and total assay times of more than an hour). This capability allowed us to decouple the timing of release of event-triggered valves from the dissolution time of the DFs (via the use of the siphon structure), but still allow the sequence of valve actuation to be determined by the disc architecture. Thus, this platform has the potential to be applied across a wider range of different biomedical diagnostic applications.

## Figures and Tables

**Table 1:** Comparison of common lab-on-a-disc valving technologies.

Name and Operation	Advantages	Disadvantages	Refs
**Capillary Valves** are actuated by increasing the disc spin-rate. They function based on the balance of body forces (governed by relative centrifugal force) and interfacial tension holding the liquid in place.	Simple operation and ease of manufacture.	Cannot operate at high disc speeds. Highly dependent on manufacturing fidelity. Low number of assay steps.	[40,41]
**Capillary-action primed siphon valves** are low-pass valves. Thy are triggered by reducing the disc spin-rate which allows capillary priming of a siphon. They can be combined in series (with capillary valves) to enable actuation by sequentially increasing and decreasing disc spin speed.	Simple operation and ease of manufacture. Can enable sample incubation.	Highly dependent on manufacturing fidelity. Low number of assay steps. Can use significant disc real-estate.	[32,42,43,44,45]
**Centrifugo-pneumatic siphon valves (CPSV)** function in a manner similar to siphon valves except the release of compressed air (trapped during loading of a reservoir) primes the siphon rather than the capillary force.	Simple operation and ease of manufacture. Can enable sample incubation. Reliable and tolerant to low-fidelity manufacture.	Can use up significant disc real-estate.	[46,47,48,49,50,51,52]
**Acceleration Actuated** valves incorporate disc features (over-flow structures, siphon valves) which are activated when the disc is rapidly accelerated or decelerated.	Highly reliable. No external instrumentation required—rotational control only.	Can require a powerful motor to generate necessary acceleration (Euler Force). Can use significant disc real-estate.	[53,54,55]
**Deformable membranes/burstable foils** are integrated into the disc during manufacture and can be tuned to open at a predetermined disc spin-rate (liquid body-forces overcomes the seal created by the membrane/foil).	Highly reliable. No external instrumentation required–rotational control only. Timing of valve actuations.	Requires additional components (integration of foils or stick-packs etc.) Complex assays limited by available motor speeds. Single use valves. Difficult to implement long incubations.	[56,57]
**Dissolvable Film (DF) (Rotational Pulse)** use integrated water-dissolvable films which are recessed into trapped gas pockets. The disc spin speed at which the liquid can be forced into the gas pocket (to wet and dissolve the DF) in inversely proportional to the size of the gas pocket. This relationship permits precise design of valve opening frequencies.	Highly reliable. No external instrumentation required–rotational control only. Timing of valve actuations.	Requires multilayer architecture. Requires embedded DF valves. Complex assays limited by available motor speeds. Single use valves. Difficult to implement long incubations.	[58,59]
**Water-clock valves** use liquid movement to sequentially vent channels which release air-locks intentionally designed into the disc architecture. This allows liquid rellease in a sequential pre-determined cascade while the disc rotates at a constant disc speed.	Sequential Valve Opening. Ease of Manufacture.	Operates only at low to medium disc speeds. Can take significant disc space. No timing of valve release. No long incubations/washes.	[39]
**Dissolvable Film (Event-triggered)** use a network of pneumatic channels which are blocked by dissolvable films. Dissolving a film at one point on the disc can trigger release of liquid through a DF located elsewhere on the disc. This allows liquid rellease in a sequential pre-determined cascade while the disc rotates at a constant disc speed.	Permits complex multi-step assays (20+ steps). Suitable for high disc spin-speeds.	Requires multilayer architecture. Requires embedded DF valves. No timing of valve release. No long incubations/washes. Single use valves.	[38,60]
**Dissolvable Film (Event-triggered with instrumentation)** incorporates the event-triggered architecture except the actuation of valves is through external actions such as piercing a tape or melting a wax film.	Permits complex multi-step assays (60+ steps). Suitable for high disc spin-speeds. Feedback control possible.	Requires multilayer architecture. Requires embedded DF valves. No long incubations/washes. Single use valves. Requires support instrumentation,	[29,61]
**Dissolvable Film (Event-triggered with Siphon Control)** are described in Figure 1	Permits complex multi-step assays. Suitable for high disc spin-speeds. Timing of valve opening/incubations using only rotational control.	Requires multilayer architecture. Requires embedded DF valves. Single use valves (except siphon).	-

**Table 2:** Reagent loading sequence. Each reservoir was loaded with 90 μL of reagent.

Assay Step	Reagent	Incubation Time (mins)	Mixing Cycles
Bead Capture (1)	0.5 mg Pre-blocked magnetic beads in Incubation Buffer	2.5	15
CRP Incubation (2)	C-Reactive protein Standard/Sample suspended in Incubation Buffer	20	120
Wash 1 (3)	Wash Solution	2.5	15
Wash 2 (4)	Wash Solution	2.5	15
Detection Antibody (5)	1:10,000 dilution in Incubation Buffer	20	120
Wash 3 (6)	Wash Solution	2.5	15
Wash 4 (7)	Wash Solution	2.5	15
Chemiluminescent Substrate (8)	N/A	N/A	N/A

## Data Availability

Videos of discs in operation are provided as part of ESI for this manuscript.

## Supplementary Materials

The following are available online at https://www.mdpi.com/2079-6374/11/3/73/s1. ESI.pdf contains additional information pertaining to the assay optimization and protocols used. Video S1: Optimized Design.mp4 shows the disc design used in these experiments. In this video, colored water was used for visualization, incubations were shortened, and the video was accelerated 8x to assist with viewing. Video S2: Dual Pneumatic.mp4 shows an earlier design used in preliminary testing. Here the centrifugo-pneumatic valve was split into two chambers to better control sample fluid height in the incubation chamber. Video play at 8x normal speed for ease of viewing.
