# Supplementary material for: Siphon-Controlled Automation on a Lab-on-a-Disc Using Event-Triggered Dissolvable Film Valves
Source: Biosensors (Basel). 2021 Mar 6;11(3):73. doi: 10.3390/bios11030073 (PMC8000095; doi:10.3390/bios11030073)
Supplement: Supplementary file 1 [file biosensors-11-00073-s001.zip › Zip2/ESI.pdf]

Electronic Supplementary Information:

Protocols:

Dynabeads Antibody Coupling Procedure: Conjugation of primary antibody to Dynabeads® M-270 Epoxy was performed as per the manufacturer's instructions (available at https://www.thermofisher.com/order/catalog/product/14301#/14301). Final concentration of primary antibody (ab31156, Abcam plc, UK) coated Dynabeads® in solution was 10 mg/mL with each mg of beads coated with approximately 1µg of antibody.

Benchtop Magnetic Chemiluminescent assay: All standards were made by diluting CRP stock (CRP Human Kit for Luminex® Platform (Catalogue number: LHP0031)) with 1X Incubation buffer (Human Extracellular Protein Buffer Reagent (Catalogue number: LHB0001)) to the appropriate concentration. 20µl of primary antibody coated Dynabeads® (10 mg/mL) were placed in each well of a white 96 well plate, using a small neodymium magnet, the Dynabeads were held in place while the liquid was removed and discarded. Each well was then blocked using a 1% solution of BSA in 0.1M PBS for 1 hour at 4°C. As before the contents of the well are discarded whilst the magnetic microparticles are held in place with a magnet. 100µL of each CRP standard was added to the corresponding well and incubated at room temperature for a given incubation time (standard incubation 2 hour). Each well and microparticle content was then washed twice with 200µL of wash solution (Human Extracellular Protein Buffer Reagent (Catalogue number: LHB0001)). 200 µL of detection antibody (PA1-28329) (1/10,000 dilution in 0.1M PBS) was added to each well and incubated for a given incubation time in the dark (standard incubation 1 hour). Each well and microparticle contents was then washed twice with 200µL of wash solution as before. Just prior to plate reading, the final wash solution was removed from each well and 100 µL of Pierce™ ECL Western Blotting Substrate (Catalogue number: 32106) was added to each well. Wells were read using a GloMax 96 Microplate Luminometer.

Lab-on-a-disk Magnetic Chemiluminescent assay: Prior to being run, each disc cartridge had two neodymium magnets placed in precut holes behind the incubation chamber, next each reservoir was loaded with 90µl of a specific reagent corresponding to a step a generic immunoassay. A total of eight reservoirs were filled as follows: reservoir 1 (R1) contains 0.5mg of pre-blocked magnetic capture antibody coupled Dynabeads in incubation buffer with 1% (w/v) BSA. R2 contains the specific CRP standard (80, 40, 20, 10, 5 or 2.5 ng/mL), R3 and R4 were assay washing solutions, R5 contained the detection antibody in 0.1M PBS, R6 and R7 contained assay washing solutions, and R8 was left empty during assay testing as the current iteration is not capable of direct luminescence reading. In future iterations this reservoir will contain HRP to trigger chemiluminescent detection. Following the final washing step (R7), the disc was stopped. The magnets were removed, and the beads were suspended in 0.1M PBS and removed, via pipetting up and down repeatedly through a vent on the incubation chamber. The magnetic microparticles were transferred to a white 96 well plate, just prior to plate reading a magnet was used to hold the Dynabeads in place while the PBS was removed. 100 µL of Pierce™ ECL Western Blotting Substrate (Catalogue number: 32106) was the added to the well and read using a GloMax 96 Microplate Luminometer.

Calculations

Limit of detection (LOD) 3.3*(SD of intercept/slope)

Limit of quantification (LOQ) 10*(SD of intercept/slope)

Video Editing

ESI Video 1 was acquired as a series of still frames which was cropped and assembled into a video format (AVI) using freely available software (VirtualDub, Irfanview). As the strobe / camera were not perfectly synchronized, the video was uploaded to Youtube (Google), a stabilizing algorithm was applied, and the video was downloaded in compressed mp4 format. Some artifacts of this algorithm may be seen in the video.

ESI Video 2 acquired as a series of still frames which was cropped and assembled into a video format (AVI) using freely available software (VirtualDub, Irfanview) and then converted into an mp4 file by Windows Movie Maker and further compressed using Windows Video Editor
